# Managing pandemics as super wicked problems: lessons from, and for, COVID-19 and the climate crisis

**DOI:** 10.1007/s11077-021-09442-2

**Published:** 2021-11-17

**Authors:** Graeme Auld, Steven Bernstein, Benjamin Cashore, Kelly Levin

**Affiliations:** 1 School of Public Policy and Administration, Carleton University, Ottawa, Canada; 2 Department of Political Science, University of Toronto, Toronto, Canada; 3 Lee Kuan Yew School of Public Policy, National University of Singapore, Singapore, Singapore; 4 Chief of Science, Data, and Systems Change, Bezos Earth Fund, Boston, MA USA

**Keywords:** Super wicked problems, COVID-19, Path dependency analysis, Climate policy, Pandemics, Health policy, Epidemiology, World Health Organization, Thermostatic institutions

## Abstract

COVID-19 has caused 100s of millions of infections and millions of deaths worldwide, overwhelming health and economic capacities in many countries and at multiple scales. The immediacy and magnitude of this crisis has resulted in government officials, practitioners and applied scholars turning to reflexive learning exercises to generate insights for managing the reverberating effects of this disease as well as the next inevitable pandemic. We contribute to both tasks by assessing COVID-19 as a “super wicked” problem denoted by four features we originally formulated to describe the climate crisis: time is running out, no central authority, those causing the problem also want to solve it, and policies irrationally discount the future (Levin et al. in Playing it forward: path dependency, progressive incrementalism, and the “super wicked” problem of global climate change, 2007; Levin et al. in Playing it forward: Path dependency, progressive incrementalism, and the "super wicked" problem of global climate change, 2009; Levin et al. in Policy Sci 45(2):123–152, 2012). Doing so leads us to identify three overarching imperatives critical for pandemic management. First, similar to requirements to address the climate crisis, policy makers must establish and maintain *durable* policy objectives. Second, in contrast to climate, management responses must always allow for swift *changes* in policy settings and calibrations given rapid and evolving knowledge about a particular disease’s epidemiology. Third, analogous to, but with swifter effects than climate, wide-ranging global efforts, if well designed, will dramatically reduce domestic costs and resource requirements by curbing the spread of the disease and/or fostering relevant knowledge for managing containment and eradication. Accomplishing these tasks requires building the analytic capacity for engaging in reflexive anticipatory policy design exercises aimed at maintaining, or building, life-saving thermostatic institutions at the global and domestic levels.

## Introduction

COVID-19 has caused 100s of millions of infections and millions of deaths worldwide, overwhelming health and economic capacities in many countries and at multiple scales.^1^ The immediacy and magnitude of this crisis has resulted in government officials, practitioners, and applied scholars turning to reflexive learning exercises to generate insights for managing the reverberating effects of this disease as well as the next inevitable pandemic (Table 1).

**Table 1:** Comparing climate and COVID-19 as super wicked problems

	*Climate*	*COVID-19*
*Time is running out*	Years and decades	Hours and days
*No central authority*	Emissions from anywhere have same global effect	Diseases spread across borders
*Those causing problem also want to solve*	People locked into, and benefit from, high carbon economy	People benefit from disease spreading social and economic networks
*Irrational discounting*	Weak commitments: pushed off as future nears	Strong commitments: risk of near term bias, moral hazard

We contribute to both tasks by assessing COVID-19 as a “super wicked” problem,^2^ denoted by four features we originally formulated to describe the climate crisis: time is running out, no central authority, those causing the problem also want to solve it, and policies irrationally discount the future (Levin et al., 2007, 2009, 2012). Doing so leads us to identify three overarching imperatives critical for pandemic policy and management. First, like the climate crisis, policy makers must establish and maintain *durable* policy goals and objectives. Second, unlike climate, management responses must always allow for swift *changes* in policy settings and calibrations given rapid and evolving knowledge about a particular disease’s epidemiology. Third, analogous to, but with swifter effects than climate, wide-ranging global efforts, if well designed, will dramatically reduce domestic costs and resource requirements by curbing the spread of the disease and/or fostering relevant knowledge for managing containment and eradication.

These conclusions expand, but are consistent with, much of the initial “lesson drawing” analyses of COVID-19 management to date. However, much less attention has been placed on identifying and developing the *analytic* capacity-building requirements necessary for achieving these management and policy imperatives (Howlett & Ramesh, 2016). We contribute to this gap by calling for greater conceptual and deliberative attention on how to build “thermostatic institutions”: that is, systems of policy interactions in which outside conditions trigger internal *changes* in some policy elements in order to *maintain stability* of policy goals and objectives (Cashore & Howlett, 2007). (Just the way a thermostat responds to changes in outside temperatures by turning on or off a furnace to maintain a house’s internal temperature.)

We elaborate this argument in the following steps. First, we compare COVID-19’s problem structure to climate change against the four features of super wicked problems. Second, we identify lessons for policy design by elaborating the three resulting policy and management imperatives. Third, we apply path dependency analysis developed for the climate case to illustrate how the presence of thermostatic institutions appeared to dramatically improve COVID-19 management, especially in the critical early days. Fourth, we show how the incorporation of these insights into anticipatory policy design requires building analytic capacity through which governments and international organizations might engage in reflexive exercises (Bali et al., 2019) aimed at maintaining, or building, life-saving thermostatic institutions at the global and domestic levels.

## The four key features of super wicked problems

Rittel and Webber (1973) identified a distinct set of “wicked” problems that stymied planners, characterized by ten features including “no stopping rule” (i.e., they lack a discrete solution or end point at which one can say the problem is solved), “no immediate test” of a potential solution, “no opportunity to learn by trial and error,” and little opportunity for a planner to be “wrong.” Critics argued that these characterizations were so broad they could describe virtually every contemporary policy problem (Levin et al., 2012; Peters, 2017). In contrast, our four features target a narrow range of particularly pernicious problems that, if well understood, would allow policy makers to avoid “ill-fit for purpose” traditional policy analysis tools such as cost–benefit analysis in favour of those that better conformed to the problem features at hand (Levin et al., 2012). Contrasting these key features derived inductively from the climate case to COVID-19 yields important insights about the similarities and differences that carry profound policy, governance, and management implications.

### Time is running out

If global average temperature increases more than 1.5 °C above pre-industrial levels, we risk catastrophic impacts to a range of terrestrial, marine, and aquatic ecosystems (IPCC, 2018). Scientists predict, based on current trajectories, there is more than a 50% chance that this level of warming is reached or crossed between 2021 and 2040 (with a central estimate of the early 2030s) (Masson-Delmotte et al., 2021). This feature also separates climate change from other important issues, such as efforts to promote universal health care or gun control. While defeats on the latter are certainly discouraging and can bring considerable human suffering, there is nothing stopping supporters from trying again in 5 or 10 years.

The time feature is even more acute for pandemics like COVID-19 because failure to take action within days or even hours, rather than years in the climate case, can risk exponential effects in deaths and illness (Sun et al., 2020). This creates a conundrum for managers faced with novel diseases who must at first, and yet when time is precious, take “best guess” efforts based on expectations rather than empirical evidence about how the disease spreads. Hence, while both COVID-19 and climate share the problem structure of time is running out, micro-level differences carry implications for how to manage them.

In both cases, scientific evidence, rather than electoral cycles or policy windows, dictates the specific time requirements. A lack of purposeful response consistent with the time dimension risks significantly greater and wider-reaching impacts. Interventions that might work early are often more challenging to implement and less effective with the passage of time. For example, extensive social contact tracing early in the spread of the virus can have positive effects but is more difficult and costly if only implemented once widespread infections occur. Likewise, the economic costs of mitigating climate change increase exponentially without action as the time becomes shorter to avoid catastrophic impacts.

### No central authority

Climate change governance is fragmented because of both the formally anarchic structure of the international system and the nature of the problem: emissions anywhere contribute, which means global action is needed. Thus, despite the long history of efforts to build international treaties and cooperation, including the 2015 Paris Agreement, addressing climate change also requires advancing transnational, multilevel, multi-scaler (Bernstein & Hoffmann, 2019), and subnational responses.

Similarly, there is no central global authority to govern pandemics. While a World Health Organization (WHO) exists, it has no power to dictate global responses or control national health agencies, policies, or regulations. Whereas the WHO performs many important functions to monitor and promote health, it primarily acts as an advisory, scientific, health intelligence, and health promotion body. Its authority is limited largely to classifying global health threats, offering advice and public health guidance, and supporting responses to essential health services in emergencies or in countries requesting assistance.

The absence of central authority measures needed to stop the disease’s spread have disproportionately affected supply chains and production that rely on global trade and commerce in general and undermined important economic sectors from aviation and tourism to food and leisure services. They also limited myriad educational opportunities and cultural interactions. Even island states like New Zealand that benefitted from a hard-line approach to limiting the spread of the disease are vulnerable to these effects.

To be sure, the ability to control borders means that national governments have more leeway to unilaterally manage the spread of a disease than they do for greenhouse gas emissions. This helps to explain decentralized responses to COVID-19 as countries relied on their sovereign authority to develop regulations on travel, border control, tracing of people within subnational and national boundaries, and domestic regulation and policing. It also explains why, in the early days of COVID-19, several countries reverted to banning the export of medical supplies and protective gear (Evans, 2020; Goodman et al., 2020; Reuters, 2021) and subsequently engaged in “vaccine nationalism”. What we do know is these uncoordinated domestic responses are ill-equipped to address a pandemic’s global dimensions that arise owing to economic, social, and diplomatic interconnectedness and associated global security challenges (Burton, 2020).

### Those seeking to end the problem are also causing it

Unlike problems with clear supporters and opponents, such as gun control or abortion, super wicked problems like climate change are characterized by those seeking to solve the problem also contributing to it: we are battling our collective selves. Virtually no one wants climate change, but the current political system, technologies, availability of energy sources, and patterns of production and consumption among other economic and cultural factors lead to us all individually and collectively contributing to the problem.

Like climate change, society is not divided among interests who support or oppose the spread, effects, and persistence of COVID-19. Yet the virus has diffused so rapidly because humans benefit from, and reinforce individually and collectively, domestic and transnational social networks, as well as economic benefits of global economic integration (Cohen et al., 2008; Gates, 2020). Accordingly, the benefits of maintaining activities that cause the problem are so strong in the short and medium terms that individuals and governments may resist changing or limiting activities that can spread disease, even if they know the risks of harm to themselves and others.^3^

### Policies discount the future irrationally

Policy responses to super wicked problems suffer from discounting the future irrationally. Economists have long recognized the phenomenon of “hyperbolic discounting” in which today’s preferences are often inconsistent with long-term economic benefits. Others have found a tendency of political institutions to “disproportionately consider certain aspects of the present” (Dietsch, 2020), especially economic over environmental values (Cashore & Bernstein, 2020), and the tendency to “put off the future” (Cashore et al., 2019; Lijphart, 1990). These “irrational” logics in turn result in policies that are inconsistent with what the scientific evidence indicates is required for addressing the problem at hand. Thus, many policies are prone to punting, identifying commitments in the future, and then reneging as the salience of short-term costs confront the intended behavioural change.

To be sure, these features play out differently given that the immediate and widespread impacts of a pandemic generate focussed attention often lacking for climate change (van der Ven & Sun, 2021). However, near-term biases are also present in the COVID-19 case. For example, politicians in Italy (Pisano et al., 2020) and the USA (Lipton et al., 2020) initially reacted sceptically to the crisis following initial advice to lock down cities and institute social distancing. While epidemiological uncertainty may have contributed to divergent responses (Yong, 2019; Landler & Castle, 2020b), some choices—repeatedly played out through various waves of the pandemic—reflect shorter-term economic and interest-based calculations (Tankersley et al., 2020; Judin, 2020; Salas & Zafra, 2020).

Moreover, the acute time pressure to act during the COVID-19 crisis also creates the risk of two additional time inconsistency challenges. First, policy makers acting swiftly risk inadvertently focussing disproportionate attention (Andrade, 2020) on a subset of impacts. Such “moral panic” (Garland, 2008) occurs when the need to act quickly fails to fully capture broader dimensions of the problem critical for long term management. A classic example is the instance of “helicopter” parenting that emerged after a handful of child abduction cases in the 1980s, which had long-term effects on the skills of a generation of children to navigate the world on their own (Odenweller et al., 2014). This phenomenon might explain, for example, why initial policy development, such as prolonged closing of schools and work places, appeared to be justified on expectations of direct benefits on human health (Quah, 2020a) rather than on equally important, but more abstract and indirect effects, such as the potential of these policies to increase the rates of domestic violence (Bradbury‐Jones & Isham, 2020), murder (Fuller & Arango, 2020), and suicide among young people (Sher, 2020).

#### Lessons for policy design

Several design lessons emerge from treating COVID-19 as a super wicked problem. First, officials must find ways to achieve policy goals and objectives that are capable of overcoming pandemic induced “irrational discounting”. Second, just as we argued for the climate case, incorporating path dependency analysis into policy design can help policy officials fend off (short-term oriented) pressures to reverse or change course (Levin et al., 2012). Third, uncovering effective policy mixes requires disentangling, and specifying, six elements: ends oriented policy goals, objectives and settings, and means oriented tools and calibrations (Table 2 and Cashore & Howlett, 2007). Doing so will help avoid “stickiness” in the wrong elements: that is, those that undermine, rather than help maintain, policy objectives. Careful attention to the specific aspects of the three management imperatives and the project of building “thermostatic” institutions serve to elaborate, and justify, design principles for effective policy mixes and global governance.

**Table 2:** Elements of policy *Source*: Adapted from Cashore and Howlett (2007: 536) and Cashore 2020

	*Policy level*		
	*High-level abstraction*	*Operationalization*	*On-the-ground specifications*
*Policy content*			
*Ends (Aims)*	**Goals** What general types of ideas govern policy development? For example, advance human health, create economic growth, social cohesion	**Objectives** What does policy formally aim to achieve? For example, saving the most lives or personal years possible, minimizing preventing economic recession, minimizing job losses	**Settings** What are the specific on-the-ground requirements? For example, # of days in quarantine, # of household visitors a day, rules government types of business that can open, and # of customers per day; level of temperature forbidding entry
*Means (Instruments)*	**Intervention Logic** What general norms guide policy instrument preferences? For example, command and compliance, hierarchy, markets, voluntary, networks	**Tools** What types of instruments are utilized? For example, regulatory tools for opening or closing business, education or financial incentives	**Calibrations** What are the specific ways in which the instrument is applied? For example, types and severity of punishment, method of distributing tax breaks or subsidies, contact tracing apps that store individual names

## Three management imperatives

### Set clear goals and policy objectives well in advance

The “time is running out” feature of pandemics requires that policy makers deliberate carefully about what goals and objectives will drive consideration of policy mixes well in advance of a pandemic being discovered. It has been widely accepted that choosing to wait until after a pandemic hits will waste precious time and most certainly risk increased fatalities (WHO, 2016; Mosk, 2021; WHO, 2021). This imperative requires deliberating carefully over the most effective policy mixes. For example, there must be a clear articulation of whether the overarching goal during a pandemic is to “save lives” or the degree to which other goals such as economic development should also influence design rationales. In the former case, economic losses matter only to the extent that they also cause lives to be lost. In the latter case, just how to weigh economic goals with lives saved will require some type of pre-determined philosophy and adjudicating framework (Cashore & Bernstein, 2020). The failure on the part of some countries to deliberate on these matters in advance of a pandemic meant that they played out in “real time”, leading to festering debates, conflict, ad hoc reversals, and inconsistent policy choices. For example, in April 2020, the T﻿exas Lieutenant governor came down on the side of economic growth by expressing his willingness to trade off his own death to save his grandchildren’s economic opportunities (Wallace, 2020), while other political leaders vehemently opposed the idea that lives were expendable (CBS News, 2020; Carter & Crawley, 2020).

For the same reasons, policy makers must be highly precise in operationalizing objectives. Projections over different mixes of policy tools, settings and calibrations—such as say, whether lock downs will be general in nature, or are targeted to, nursing homes - will have different effects on whether the objective is to “save the most lives” or “reduce the amount of personal years lost” (Layard et al., 2020).

Failure to deliberate in advance over what goals and objectives ought to guide policy mixes wastes valuable time and means policy makers will be more vulnerable to pressures to change goals and objectives as the super wicked “irrational discounting” feature takes hold (Stone & Gray, 2020). It also makes it more likely that values held by experts, rather than their knowledge of epidemiology, will end up influencing these choices through the back door (Porter, 2020). For example, the head of the University of Oxford’s Evolutionary Ecology of Infectious Disease group, Sunetra Gupta, controversially contradicted much of the prevailing epidemiological modelling by positing that restrictive quarantines were not needed (Sayers, 2020; Cookson, 2020). However, a careful reading of Gupta’s analysis reveals that these differences were owing to her personal values, rather than her epidemiological expertise, that policy mixes recommendations had a duty to incorporate *inequality* effects of quarantines (Sayers, 2020: italics added). Rendering these explicit would allow for society and government officials to deliberate, in advance, over how to adjudicate competing objectives and/or whether they can be made complementary.

### Maintain objectives by facilitating changes in tools, calibrations and settings

Second, durable goals and objectives must allow for the potential of changes in other policy elements in response to evolving epidemiology (Brueck & Wyman, 2020; Roberts, 2020). While climate policy mixes can foster “sticky” policy settings that well established scientific research indicates are consistent with reducing the problem at hand—such as coercive settings requiring cycling over automobile use that, all else being equal, will lower carbon emissions—initial pandemic management affords no such luxury because such established science is lacking. This poses a conundrum: the “time is running out feature” requires that action be taken immediately in hours or days, while in the initial stages of a pandemic the least is known about how the disease operates. For example, initial temperature screening at airports based on SARS epidemiology did not conform to subsequent evidence of asymptomatic spread of COVID-19. Practically this means engaging in “best guess” policy mixes that avoid “sticky” settings, tools, and calibrations that make it difficult to expand or pivot to more effective approaches, such as mandatory two-week quarantines or mask wearing.

The “ability to mount fast and adaptable responses” (WHO, 2021: 19) may seem obvious, but widespread evidence reveals that policy designers made choices that locked in settings and calibrations that hindered an ability to make swift changes. For example, one of Thailand’s initial policy mixes required the installation of foot operated elevators when the early epidemiology suggested that the virus spread easily via surfaces (Strait Times, 2020b). When subsequent research challenged these expectations (Fortin, 2020), sunk costs in this initial investment made it difficult and impractical to reverse course—resulting in “locking in” scarce resources that would have been more effective if applied elsewhere. Likewise some private sector housing developments are considering adapting their 100-year urban renewal projects based on the COVID-19 epidemiology (Hipwood, 2020) when there is no guarantee, as illustrated in the Thai elevator or the airport temperature cases, that such redesigns will produce behaviours to minimize the effects of the next pandemic.

Similarly, and arguably most troublingly, some governments appeared to become hostage to their initial “best guess” decisions about their choices of policy mixes, even when emerging epidemiology produced evidence inconsistent with their cause-and-effect projections. For example, the Swedish government initially took a largely voluntary approach to policy settings and calibrations that stood it apart from its Scandinavian neighbours. It based this approach on expert projections and incorporated the science of “herd immunity.” However, over time, even as evidence indicated that Sweden’s death rate was higher than its Scandinavian neighbours Denmark, Norway, and Finland (Goodman & Carmichael, 2021), it “doubled down” (Claeson & Hanson, 2021) on its existing policy settings, tools, and calibrations (Rolander, 2021; The Guardian, 2020). Instead of reversing course, the government “[downplayed] the roles of asymptomatic spread, aerosol transmission, children as potential sources of infection, and the use of face masks” (Claeson & Hanson, 2021).

What is important for our analysis is the need to foster the analytic capacity to unpack and avoid the specific causal factors at play that lock-in the wrong policy elements. For example, the Thai foot elevator and architecture projects were sticky owing to fixed cost infrastructure investments. Others have argued that the Swedish example was owing to the role of public opinion, “face saving” of elected officials, and bureaucrats’ “stick-to-it-iveness” tendencies (Claeson & Hanson, 2021). However, in other cases, stickiness can be traced back to administrative dynamics, such as time lags associated with developing new policy tools, regulations, and technological advances to change initial practices resulting from early policy mixes (WHO, 2021: 33). The precise set of factors matter given they help assess whether, and how, changes in policy settings, calibrations and tools might occur. For example, in contrast to fixed costs of investment or face-saving explanations, reducing administrative time lags may be relatively easy to overcome by targeting policy settings and steering activities to those that are relatively easily ramped up and down—such as regulations about whether employees are required to work from home or the office or supporting online and delivery services.

While changes in settings, tools, and calibrations must be facilitated to achieve durable objectives, it is equally notable that changes owing to confusion and vacillation about goals and objectives must be avoided. For example, a second wave of infections in Ontario, Canada in fall 2020 led to a new policy mix that included a colour-coded system of graduated restrictions on businesses and social gatherings based on daily rates of infection. Yet the precise settings were less stringent than those in the first wave in spring 2020, allowing gathering four times higher than those recommended by its own public health agency. In contrast to its earlier exhortation that lives were not expendable, the government defended these changes not based on emerging epidemiology, but, reflecting the tendency of super wicked problems to produce irrational policy decisions, an explicit effort to balance pandemic decisions with economic goals (Stone & Gray, 2020). Later, in response to knowledge about new variants in April 2021, Ontario appeared to reverse course by announcing heightened lockdowns, including closing playgrounds and shutting provincial borders to non-essential travel (Willms & Atalick, 2021). It simultaneously changed settings to allow more business to open while formally rejecting policy recommendations for paid sick days aimed at keeping individuals isolated. These inconsistencies in turn, appeared to undermine the public’s trust (Ferguson & Benzie, 2021).

### Build influential global governance

The need to respond to the “lack of central authority” feature of super wicked problems has been long recognized by governments and experts, with “at least 11 high-level panels and commissions [making] specific recommendations in 16 reports to improve global pandemic preparedness” since the 2009 H1N1 epidemic (WHO, 2021: 19). There have also been repeated calls for increased funding for WHO to improve its ability to lead and coordinate swift responses to international health emergencies (Gostin et al., 2020).

Yet despite these recommendations, global pandemic architecture has gone in the opposite direction. For example, the WHO (2021) high-level independent panel on pandemic preparedness notably lamented the “conservative” 2005 International Health Regulations (IHR), which governs the current alert system for international health emergencies, “does not operate with sufficient speed when faced with a fast-moving respiratory pathogen ….” that as a result “serve to constrain rather than facilitate rapid action” (WHO, 2021: 26). The panel also noted that this was owing, in large part, to the current IHR focus on *limiting* “unnecessary” restrictions on travel and trade and *increasing* requirements before a health emergency can be declared compared to earlier versions. Recognition of the “time is running out” feature of super wicked problems led the panel to lament the undermining of WHO’s autonomy and its ability to initiate a rapid and precautionary approach (WHO, 2021: 26). This extant environment hampered the ability of the WHO to swiftly adjust communication tools, including associated settings and calibrations, that would have greatly assisted country-level responses to COVID-19 (WHO, 2021: 24–26). The result was February 2020 being “a lost month, when steps could and should have been taken to curtail the epidemic and forestall the pandemic” (WHO, 2021: 19).

## The institutional building blocks for pandemic management

### Path-dependent thermostats

All three management imperatives require maintaining or building what Cashore and Howlett (2007) refer to as “thermostatic institutions” denoted by two related dynamics: (a) the automatic triggering of swift *changes* in one or more policy elements following some type of “external perturbation” in order to maintain *durability* of other elements; (b) a high degree of *durability* to withstand (short-term) political and other pressures to eliminate the thermostatic institution itself. Building such thermostats in the COVID-19 case would, if successful, produce changes in settings to maintain policy objectives and overcome irrational discounting. Such thermostats at the global level would also be expected help achieve the longstanding consensus that pandemic management requires a global “system that can adapt and correct itself” (WHO, 2021: 27).

A long history of social science research includes examples of thermostats triggered by policy (Hacker, 1998) and organizational (Jinnah, 2014; Levi-Faur & Jordana, 2006; Valdés, 2011) reforms. For example, US endangered species policy—from which Cashore and Howlett (2007) first derived the thermostatic concept—required that, once a species was listed as endangered, a federal agency must develop a management plan capable of maintaining its “viability”. This meant that when scientific evidence indicated that logging practices in old growth forests threatened a specific species with extinction, massive *changes* in settings, tools, and calibrations occurred that increased conservation outcomes in order to *maintain* policy objectives. (Timber industry and congressional delegations were unable to dismantle this thermostat owing to its path-dependent status.)

The most prominent organizational example is the creation of relatively autonomous central banks across most countries (Jordana & Rosas, 2014) that shield monetary policies from short-term political pressures and/or other policy problems in pursuit of goals like price stability. This allows these organizations to freely adjust settings (e.g. target interest rates) and tool calibrations (e.g. lending and borrowing money via different mechanisms) to maintain long-term durable objectives such as inflation targets and exchange rates (Pollitt et al., 2004).

### Path dependency analysis: lessons from climate

How then, might policy designers innovate to create path-dependent thermostats for pandemic management? Our application of path dependency analysis to climate change offers a promising line of inquiry with which to develop and apply requisite analytic skills. We identified two central analytic steps for fostering such “anticipatory policy design.” First, we identified diagnostic questions for overcoming one or more of the four features of super wicked problems: what has or can be done to create immediate policy stickiness or irreversibility? (DQ1); what has or can be done to entrench support over time? (DQ2); and what has or can be done to expand populations that support the policy over time? (DQ3). The main logic undergirding the questions is that initial policies must be put in place that could generate trajectories designed to overcome one or more of the four features of super wicked problems (Table 3). Although implied in earlier work, we have since added a fourth diagnostic question to avoid application of proposed approaches inconsistent with explicit policy objectives: what has or can be done to ensure that lock-in, entrenchment, and expansion initiatives are in line with desired outcomes? (DQ4).

**Table 3:** Diagnostic questions most relevant for specific super wicked features *Source*: adapted from Levin et al. (2012)

	Time is running out	No central authority	Those causing wanting to solve	Irrational discounting
DQ1: Immediate stickiness	✓		✓	
DQ2: Entrenched over time	✓		✓	✓
DQ3: Expanding population		✓	✓	
DQ4: Required outcome	✓			✓

Second, we turned to identifying how to purposely trigger historically contingent “critical junctures” (Capoccia & Kelemen, 2007; Lockwood et al., 2017) capable of fostering multiple step causal processes that would be expected, over time, to shape future politics and institutional authority and foster thinking about their role in shaping social movements, political parties, and norms that define appropriate policies or processes (Cashore & Howlett, 2007; Hacker, 2001). This led us to apply four distinct causal processes policy designers might unleash (Auld, 2009; Mahoney, 2000; Page, 2006; Pierson, 2004). *Lock-in* emphasized immediate durability owing to such factors as high fixed costs or institutional rules requiring super majorities to reverse. *Increasing returns* assessed how benefits to citizens, interest groups, and society might change over time. S*elf-reinforcing* considered whether the costs of reversal would likely increase over time due to job skills or social practices, for instance, becoming routinized and taken for granted. *Positive feedbacks* considered policies that gained support of new populations, thus expanding coalitions or popular support, while reinforcing, rather than undermining, support of the original populations. We and others have also identified the need to devote attention to *undermining* effects that any policy design can be expected to simultaneously unleash (Sewerin et al., 2020).

This approach reconciles the burgeoning but contradictory recommendations on COVID-19 highlighted by Ansell et al.’s (2020) call to “make public institutions and programs more *flexible and agile* so that they can transform and adapt themselves in response to turbulence and scale their problem-solving efforts up and down” with Green’s exhortation (2020) that COVID-19 management requires fostering policy lock-in by taking advantage of this historical “*critical juncture.*” Likewise, path dependency analysis is well poised to be applied *backward* to understand and explain historical path-dependent trajectories and *forward* to avoid running poor experiments by developing innovative policy mixes that are capable of triggering path-dependent effects.

### Path dependency analysis of COVID-19 management: looking backward for thermostats

#### Positive illustrations

Several countries—especially in Southeast Asia—already appeared to have in place thermostatic institutions relevant for pandemic management (Quah, 2020a; Hille & White, 2020) designed to quickly initiate specific settings meant to reduce the risk of infection. For example, Singapore’s experience with SARS (DQ2) led it to immediately invoke an “interagency” committee with a designated command structure to create a quick and efficient response to COVID-19 (Woo, 2020; Quah, 2020b). Similarly, Korea’s central government had, following its experience with MERS (DQ2), specifically adopted a set of institutionalized protocols designed to allow it to realize its explicit objectives of avoiding as many deaths as possible through disease management. These designs included the "Korea Centres for Disease Control" which was granted increased epidemiological capacity and policy making autonomy (DQ4). The presence of these domestic coordinating efforts, developed well in advance of COVID-19, correlated with relatively efficient and proactive changes in policy settings, tools, and calibrations to manage COVID-19 (Evans, 2020; Quah, 2020b; Capano et al., 2020). For example, all three countries quickly initiated temperature screenings and travel restrictions and quarantines, employment of thousands of enforcement officers, and introduced high levels of fines and incarceration for non-compliance (Goddard, 2020; Ha, 2016).

Important for our analysis, these institutionalized coordinating efforts were not only designed with the thermostatic features consistent with the three imperatives for pandemic management, but also for the durability of the thermostatic institution itself (DQs1&2). For example, Korea designed its pandemic approach to policy development to avoid the panic and confusion among citizens (DQ3) that might undermine its legitimacy (Ha, 2016). It did so through several built-in measures aimed at fostering information transparency (DQ2), with the expressed rationale that doing so would facilitate the need to constantly change specific settings and calibrations as epidemiology evolved. Specific measures included the rapid release of statistics on infections and fatality rates, detailed information on locations of new infections, and government apps that allowed individuals to track where infected patients had visited (Moon, 2020). Korea’s approach to drawing on transparency as a tool for managing pandemics also seems sensitive to both reinforcing (DQ3) the thermostat itself and to avoiding undermining effects. This led government officials to consciously take into account privacy concerns that otherwise might have reduced public support for policies emanating from the thermostatic institution (DQ2) (Moon, 2020).^4^

The Korean approach also required the triggering, and charging, of an interdisciplinary scientific knowledge committee made up of economists, sociologists, and infectious disease experts to develop public guidelines on social distancing consistent with emerging science of the virus. This not only conformed to management imperatives #1 and #2, but also to fostering the durability of the thermostat itself to the extent that the expert panel enhanced public trust and legitimacy (DQs2&3). Taken together, this design allowed relatively quick adaptations of tools and calibrations that engendered public support, such as reducing stress on hospitals by using public facilities to house infected patients with mild symptoms during their quarantine or setting up drive-through and walk through testing (DQs 2, 3 &4) (Moon, 2020).

#### Negative illustrations

Other domestic cases appeared less thermostatic. For example, the UK’s approach to its “Scientific Advisory Group for Emergencies” (SAGE) left ambiguous on what types of science SAGE would base its advice (Landler & Castle, 2020a). Even more importantly, it failed to specify exactly what health goals and objectives it would pursue, or how it would handle countervailing economic and health considerations (Landler & Castle, 2020a). Partly for these reasons, SAGE faced strong public criticism for its secrecy and its initial approach to settings aimed at fostering “herd immunity.” When epidemiological science indicated that more lives would be saved by “flattening the curve,” SAGE became the target of further public criticism, and it appeared unable to account for, and adjust to the latest science (DQ4). All these dynamics appear to have undermined rather than entrenched (DQ1 and 2) public support.

Likewise, in the USA, the Centres for Disease Control and Prevention (CDC), the Food and Drug Administration and the National Institute of Allergy and Infectious Diseases all have some policy making authority, which at times produced contradictory guidelines. The US administration eventually addressed these issues through efforts to build a coordinated “command structure,” but unlike Korea and Singapore, only after COVID-19 had already started to wreak its havoc (Cancryn et al., 2020). These developments arguably played a role in creating confusion and uncertainty about the objectives driving the pandemic response. And, in contrast to thermostatic features of autonomy like that of central banks, the ad hoc institutional responses worked to foster shifting political leadership and perceived interference in the task force’s work. Resulting “real time” decisions to appoint an experienced public health expert to coordinate interagency approaches did reflect implicit attention to DQs 2&3 (Wright, 2021), but failure to develop institutional designs before the pandemic hit led to delayed changes to settings and tool calibrations. This, in turn, resulted in more lives lost (DQ4) than if the easily anticipated need for greater coordination had been recognized before COVID-19 (Jewell and Jewell 2020). This lack of coordination may explain why epidemiological experts often turned to advocacy and even op-ed writing as a way to convey scientific findings, and why the Trump administration would often critique the scientists on whose knowledge a well-functioning thermostatic institution would need to draw (Strait Times, 2020a).

Similarly, debates about whether to treat COVID-19 as an ongoing health emergency with features of a “super wicked” problem requiring efforts to constrain and direct our future selves (DQs 1, 2, 3 & 4), versus as simply a temporary impediment to economic growth goals or electoral success, arguably played a role in undermining the effectives of, and compliance with, COVID-19 related settings and tool calibrations. For example, Trump’s decision early on to strike another committee of experts designed to “re-open the economy,” made up primarily of business leaders, further exacerbated confusion about policy objectives (White House, 2020). Comparing the positive and negative cases also points to the importance of locking in, in advance, coordinating bodies “at the highest levels” (WHO, 2021: 18–20) with clear goals and objectives. This means shifting from reinforcing expected conclusions that “[e]ffective and high-level coordinating bodies were critical to a country’s ability to adapt to changing information” (WHO, 2021: 31) to instead identifying policy designs for achieving these imperatives.

## Conclusion: policy analytic capacity for building thermostats

Three principles emerge from our analysis with which to guide the development of policy analytic capacity capable of achieving the three policy and management imperatives.

### #1: Recognize two distinct, but related, path dependency tasks

#### Authority

First, the relevant global governance institutional arrangements or domestic bureaucratic agency or interagency committees charged with developing pandemic relevant policies and procedures must be designed in a way that is expected, throughout the course of the pandemic, to create path-dependent authority while avoiding undermining effects. This task is especially relevant to the design of global pandemic governance (management imperative #3) and for those domestic policy officials where there was initially no clear organizational body charged with pandemic management and/or in which societal trust and legitimacy receded, rather than expanded, over time.

Designing for these features requires drawing on analytical tools from political science and public administration that seek to understand the conditions through which political legitimacy and trust might reinforce domestic and global authority to create rules and procedures. This literature is generally motivated to understand the world not simply by how public and interest groups react to policy decisions, but also how and whether constituencies view the arenas that promulgate the rules as legitimate or appropriate for constraining or directing individual and collective behaviour (March & Olsen, 1998).

In the case of global health, this focus is important for understanding why different sources of information are perceived as more legitimate in different international health crises and the conditions that generate or undermine trust in domestic and international institutions including the WHO (Davies & Wenham, 2020). Consider one example. Most assessments agree that the WHO did receive and transmit information on COVID-19 in a relatively timely fashion. However, trust in the WHO and its advice was hard to build given persistent concerns about its conservative decision-making procedures and the perception of political interference by China (Davies & Wenham, 2020). Hence, it is critical that future anticipatory policy deliberations designed to avoid, rather than repeat, mistakes made during COVID-19 incorporate scholarship on trust and legitimacy. Doing so is especially important for maintaining support for the relevant thermostatic institution itself, even when a particular constituency may be opposed to, or negatively affected by, a particular policy choice at any given time (Suchman, 1995).

#### Objectives

Second, management imperatives 1 and 2 require that policy decisions across all six elements must be projected to foster path-dependent objectives and permit the adaptation of settings, tools, and calibrations consistent with evolving evidence. This is easier said than done. The “time is running out” feature of super wicked problems means there is no luxury of engaging in “trial and error” approaches to assess what works. Proposals for reforms, therefore, must carefully project their impacts forward in ways that account for the myriad variables at play and the societal and institutional bottlenecks expected to impede actions. It also requires deliberating over, being aware of, and avoiding as much as possible, designs where advancing path-dependent authority may undermine path-dependent policy objectives, and vice versa.

### #2: Foster forward looking deliberative causal assessments

How then might post COVID-19 “lessons learned” exercises be fostered that directly incorporate the three management imperatives and the need to create durable thermostats and policy objectives? This is not a straightforward matter of simply “carbon copying” policy settings and calibrations or specific institutional designs from countries such as Singapore and South Korea that had initial successes in curbing the spread of the disease. Careful attention to different political systems, historical developments, structural conditions or institutional legacies will be required when building, or maintaining, path-dependent thermostats and objectives (Davies & Wenham, 2020). Hence, the most important lesson is that policy recommendation exercises must move from a largely ahistorical emphasis on substantive and universalist policy recommendations to approaches that incorporate knowledge about how to trigger “critical junctures” within specific domestic and global contexts.

Doing so will open opportunities to entrench path-dependent institutions well before the next pandemic. We suggest that four steps will help guide this incredibly complex task:

1. Begin with an overarching proposal for building path-dependent institutions and objectives. Be sure to expand, rather than narrow, the kinds of creative design thinking needed for moving forward (Cashore & Bernstein, 2020). This requires incorporating insights from several disciplines, while being aware of, and guarding against being influenced by, their extant ontological and methodological biases (Grabs et al., 2020)
2. Project as many impacts as possible. Such exercises may draw on, but be cautious about, scenario building and quantitative modelling efforts in which (often hidden) assumptions about causal processes, can create false expectations of certainty (Dean, 2020; Blackwell, 2021). This effort must also attend to possible simultaneous inverse relationships between interventions that might enhance authority, such as trust generating exercises—for example information sharing initiatives that inadvertently highlight some problems over others—that end up creating simultaneous undermining effects on the durability of goals or objectives—such as, say, greater knowledge about vaccine side effect vis-à-vis their life saving impacts.
3. Engage in “back and forth” reasoning among a diverse set of knowledge holders. This requires considering, projecting, re-designing, projecting again, and redesigning such that the ultimate proposal is deemed to have a “plausible causal” logic that it can create pandemic relevant domestic and global thermostatics while minimizing undermining effects. This step must also distinguish between the institutional or structural “logic” embedded in the design, as well as the agency officials can exercise to reinforce, or nurture, a path-dependent trajectory (Cashore et al., 2019).
4. Formally document the design and causal expectations. This is because the logic for creating the institution or policy decision needs to be fully understood and shared by individuals and organizations. This shared knowledge will assist officials charged with implementing the plans in nurturing forward trajectories that enhance institutional authority and durable policy objectives.

### #3: Eschew path-undermining expert “stock taking” exercises

It is telling that pandemic-related reforms over the last 20 years have generally gone in the opposite direction than what has been recommended by a steady stream of expert “stock taking” exercises. These deliberations have compounded the problem by identifying reforms derived from post hoc analyses of the last crisis that fail to systematically take into account complex historical processes in which specific management challenges are located. In addition, they almost always bypass considerations of the undermining and reinforcing effects of their recommendations in building thermostatic institutions.

Consider, for example, the recommendations from the latest WHO expert analysis which failed to recognize that the IHR (2005) reforms—far from simply being misguided—resulted from strongly path-dependent global *political* responses to the 2003 SARS outbreak. Incorporating these historical and geo-political dynamics would have led to recognizing that it was post-SARS politics that explain the critical juncture “paradigm shift”—as the WHO labelled it (WHO, 2007)—that pivoted away from border controls to “containment at source.” The former were criticized at the time for imposing punitive travel and trade restrictions that simultaneously undermined economic development and created perverse incentives to limit information sharing about disease outbreaks (Ferhani & Rushton, 2020: 462–466). Recognition that it was these political dynamics following the SARS epidemic that *locked in* a conservative decision-making process would have been required to make recommendations for overcoming, rather than ignoring, this type of bottleneck.

These path-dependent dynamics would have also placed in context debates about whether China held back information from WHO in December 2019 and early January 2020. Regardless of the veracity of these claims, we do know that Chinese experts publicly confirmed human-to-human transmission prior to the WHO Director-General convening a first meeting of the WHO Emergency Committee on January 22, 2020. The committee also knew about confirmed cases in Thailand and Japan. Yet the conservative IHR guidelines meant that it took until a second meeting on January 30—during which time the virus had spread to 18 countries outside of China—to achieve a consensus allowing the Director-General to declare a “Public Health Emergency of International Concern” (PHEIC), the highest level of global concern under the IHR. Such an approach would have helped explain why emergency declaration thresholds led to cautious communication and a recommendation *not to* impose border controls. In fact, attention to the precise wording of policy settings, tools, and calibrations would have also explained why the WHO did not declare COVID-19 as a “pandemic” in its PHEIC declaration. This omission, which was widely criticized as fostering misunderstanding of the threat or appropriate response measures by many governments (WHO, 2021), was actually because the IHR lacked careful attention to operationalizing and defining what, precisely, constituted an actual “pandemic.” Hence, expanding attention from a focus simply on information, to including bureaucratic procedures and cascading causal processes in decision-making and implementation, would have been needed to both assess the slow global response to COVID-19, as well as how to design “forward” to avoid such outcomes for future pandemics.

To be sure, our aim here is to pave the way for, rather than adjudicate, existing debates about the pros and cons of specific policy recommendations. For example, enhancing path dependency analysis capacity would reorient debates over the value of border restrictions (Ferhani & Rushton, 2020; Kenwick & Simmons, 2020; Mallapaty, 2021) to explicitly incorporate causal assessments into how specific proposals might reinforce, or undermine, the creation and operation of durable thermostats and policy objectives. This would require incorporating positive and negative impacts of border controls—from direct effects of slowing the spread of a disease to unintended *consequence*s, such as discriminatory limits on immigration and human rights abuses, disruptions to supply chains for necessary health supplies, or the undermining of international cooperation (Ferhani & Rushton, 2020; Kenwick & Simmons, 2020).

The same approach would be needed to detail and project forward creative ideas for the design of new global institutions for pandemic management to take advantage of the “critical juncture” window the world is now presented. For example, the 2021 WHO independent panel, consistent with our three management imperatives, recognizes the importance of high-level political consensus on goals and objectives and authoritative global governance. In fact it recommends the creation of a high-level “Global Health Threats Council” or similar body (WHO, 2021). However, given the history of the failure of governments and intergovernmental processes to respond to recommendations from this type of “stock taking” exercise, it seems reasonable to conclude that much more systematic attention to the development and application of appropriate policy analytical capacity—specifically path dependency analysis—may be expected to produce more sophisticated designs for achieving, rather than calling for, these reforms.

Finally, we must note that attention to treating pandemics as super wicked problems also carries lessons for our earlier work on the climate crisis. Climate governance can also benefit from greater analytical attention to the way specific policy mixes—given an increasing reliance on “goal based” promises such as “net zero by 2050”—might produce “thermostatic” institutions capable of maintaining rather than—as we have witnessed from the last 30 years of climate governance—avoiding these commitments as time marches on.

What is clear from our analysis is that the devastating effects of COVID-19 have presented the world two doors: one in which we can decide to collectively learn from, and generate insights for, managing super wicked problems; or one in which we can repeat the design mistakes of the past.

## Footnotes

1 Mosk (2021).

2 Characterizations of COVID-19 as “super wicked” have already appeared in the media (Osaka, 2020), scholarly opinion pieces (Elkington, 2020; Boin et al., 2020) as well as special issues of journals (McConnell & Stark, 2020; Sahin & Richards, 2020).

3 To be sure, the poor and vulnerable contribute less to climate and pandemic challenges, but generally suffer more (Aubrey & Neel, 2020; Abi-Habib & Yasir, 2020).

4 An April 2020 survey found that 74.4% of respondents agreed that the government was being transparent and 60% indicated they trusted government officials that, Moon (2020) argues, helped foster public support for MER’s entrenched “agile and adaptive” approach that facilitated swift changes in policy tools, calibrations and setting, including walk-through and drive-through testing and detailed contact tracing.
